# A framework for reinitiating global academic exchange in the context of the COVID-19 pandemic

**DOI:** 10.5116/ijme.62fa.0af4

**Published:** 2022-09-09

**Authors:** Caitrin M. Kelly, Fatma Some, Daniel A. Guiles, Matthew Turissini, Adrian Gardner, Debra K. Litzelman

**Affiliations:** 1 Department of Medicine, Indiana University, Indianapolis, IN, USA; 2 Internal Medicine, Moi University School of Medicine, Eldoret, Kenya; 3 Department of Medicine and the Department of Pediatrics at Indiana University, IN, USA; 4 Indiana University Center for Global Health, Indianapolis, IN, USA

**Keywords:** Covid-19 pandemic, medical education, academic exchange, global health partnerships, travel

## Introduction

The rapid spread of SARS-COV-2 infections and the World Health Organization (WHO) declaration of a global pandemic in March of 2020 led to widespread border restrictions, closures of educational institutions, and recalling of students and employees from abroad.^1^ Following the guidance by the United States (U.S.) Centers for Disease Control and Prevention (CDC) and the U.S. Department of State, universities and health systems instituted restrictions on international travel during the COVID-19 pandemic and global health partnerships largely discontinued the exchange of faculty and trainees.^2^  This hiatus in travel triggered a paradigm shift in global health, fostering virtual innovations and dramatic growth in online education and training, telemedicine, and videoconference-based collaborations.^3^^-^^7^ Virtual global collaborations are laudable for reducing inequities stemming from unidirectional travel from the global North to the South, decreasing the carbon footprint generated by international flights, enabling broader faculty involvement in global health initiatives, and creating greater access to educational opportunities across borders.^4^^-^^6^  This paradigm shift presents an opportunity to further the decolonization of global health by redirecting travel funding to support capacity building in low and middle-income countries, with remote and more cost-effective engagement by partners in high-income countries.^3^^,^^6^^,^^8^ While online collaboration offers clear benefits, many recognize that virtual exchanges fail to build partnerships based on interpersonal relationships and an in-depth understanding of context.^4^^,^^5^^,^^7^ In addition, synchronous video-conferencing can be limited by poor bandwidth, connectivity issues, power outages, and time-zone differences.^4^^,^^7^ Global health in the post-pandemic era is likely to become a hybrid model, with longitudinal virtual collaborations supported by more limited in-person exchanges. The challenge for institutions remains how to reinitiate in-person exchange safely and equitably across international borders in the face of an ongoing pandemic complicated by new strains of COVID-19.

The purpose of this perspective piece is to describe the pandemic travel framework developed by Indiana University (I.U.) and Moi University (M.U.) as part of the Academic Model Providing Access to Healthcare (AMPATH) for academic institutions grappling with the challenge of resuming in-person international exchanges. AMPATH is a network of academic health centers collaborating with host academic institutions to support holistic, sustainable healthcare systems around the world. AMPATH-Kenya, the first AMPATH site, is an alliance between Moi Teaching and Referral Hospital (MTRH), M.U., and the AMPATH Consortium, a collaboration of universities and academic health centers led by I.U. Descriptions of clinical, education and research exchange over 30 years have been previously published.^9^^-^^14^ The Indiana University Center for Global Health (IUCGH) pandemic travel framework outlines travel logistics, institutional considerations, and pandemic safety with specific criteria that is reviewed on a weekly basis. The framework was initially developed by consensus of stakeholders from Indiana

University and Moi University for travel from the United States to Kenya but has subsequently been adapted for broader international travel applications.

The first section of the framework details pandemic travel logistics and accommodation requirements. The availability of international flights and permission for non-citizens to enter the foreign territory constitutes the first criteria. The next criteria stipulates the absence of a mandated quarantine for asymptomatic travelers with pre-travel COVID testing, or if required, the availability of safe and comfortable quarantine accommodations. Travelers must agree to comply with all COVID-19 testing requirements for entering the foreign country and re-entering the U.S., and COVID-19 testing suitable for travel must be available. Prior to vaccination being mandated by the Government of Kenya, our program required that visitors with clinical engagements be fully vaccinated, with exceptions for non-clinical visitors considered on a case-by-case basis. Specific housing units on the AMPATH consortium housing complex were designated for quarantine and isolation with other rooms limited to single occupancy. Food services on the AMPATH consortium housing complex were restructured to enable socially distanced dining in outdoor areas. A parameter was created to encourage travelers to consider how their individual health and personal circumstances might impact medical risk and mental well-being during a pandemic. Travelers are required to submit a risk acknowledgment form and approval request form derived from the described framework for review by the Associate Dean for Global Health.

Given limitations to vaccine efficacy and the continuously emerging COVID-19 variants, IUCGH included criteria to consider ongoing pandemic health risks due to a new surge. During COVID-19 peaks, hospital beds may become limited in Kenya, which impacts access to emergency and critical care services. Data on the number of new COVID-19 cases and percent positivity are published on a regular basis by the Government of Kenya and tracked by IUCGH. We estimate critical care and COVID-19 bed availability using professional and personal networks, communicating with individual medical providers at different hospitals via personal connections built through three decades of collaborations. Locally adapted COVID-19 exposure, quarantine, isolation, home-based care, and hospitalization procedures were developed for the program based on Kenya Ministry of Health, WHO, and CDC guidelines. These protocols for the AMPATH consortium housing complex are overseen by an I.U. internal medicine faculty member based in Kenya. All visitors are required to obtain evacuation insurance, and local insurance purchased through the African Medical and Research Foundation (AMREF)15 to cover emergency transportation to Nairobi in case of severe COVID-19 infection. Individuals are expected to cover any costs incurred by quarantine and COVID-19 testing. Short-term clinical visitors are required to carry their own supply of scrubs, hand sanitizer, reusable goggles, and masks.

Significant challenges in the implementation of the framework arise from new COVID-19 variants of significance. New virus strains have triggered sporadic lockdowns and rapid changes to pre-travel COVID-19 screening requirements. Although a routine review of governmental websites can prevent issues, some travelers have been required to delay or re-route travel to comply with unexpected policy changes. Our program rapidly communicates any pandemic concern or change in travel policies through a pre-existing group text platform created for emergency preparedness. Additionally, formidable challenges arise from the timespan between advanced academic scheduling, travel preparations and dynamic COVID-19 surges. IUCGH grants preliminary travel approvals but then continues to reassess feasibility and risk until a few weeks prior to scheduled travel. All short-term travelers and trainees are expected to make alternative plans and are approved for travel with the understanding that it may be rescinded in response to changing circumstances. Travel is now feasible, but the dynamic nature of the pandemic requires flexibility and frequent communication across institutions.

There are notable limitations to the framework described. Overall, it was developed for travelers from the United States to Kenya, and criteria may need to be modified for other international exchange programs. The resumption of Kenyan partners and trainees traveling to the U.S. has been delayed due to logistical challenges. Initially, limited vaccine availability and the prolonged closure of academic institutions in Kenya eliminated the elective time when M.U. trainees had the opportunity to participate in away-rotations at AMPATH institutions. Furthermore, limited operations of the U.S. Embassy in Kenya during the pandemic created a backlog of visa appointments, resulting in ongoing administrative barriers for Kenyan partners to travel to the United States. These logistical issues may negatively impact the equity in bidirectional exchange for several years. Finally, the framework was not formally validated, although it has been regularly modified to reflect changing pandemic circumstances and policies and has guided the operations of our program successfully since it was created in 2020.

The COVID-19 pandemic provides an opportunity to reflect critically on approaches to achieving bilateral institutional and individual objectives in the context of restricted travel.   The application of this framework requires an in-depth understanding of the context of the local health system and political environment. Our program has been able to make informed decisions about travel because the Kenya Ministry of Health regularly shares data on the number of new COVID-19 infections, percent positivity, vaccinations administered, and deaths. We are able to verify these trends and other vital information such as testing availability, hospital bed capacity, and nosocomial transmission by leveraging professional networks in Kenya. Transparency on the state of the pandemic from the national level, as well as ongoing communication with institutional leaders is fundamental. Prolonged suspension in international exchanges may threaten the viability of global health partnerships and lead to the languishing of counterpart relationships and initiatives. The described framework provides a prototype to evaluate the feasibility, acceptability, and safety of reinitiating in-person exchange for global health partnerships in the context of a pandemic.  

### Acknowledgments

The authors would like to thank Dr. Laura Ruhl, Dr. Jenny Baenziger, Dr. Julia Songok, Victoria Eder, and Megan Miller for their contributions to the development of the COVID-19 travel framework and institutional approval processes described.

## Conflict of Interest

The authors declare that they have no conflict of interest.
